# Record high Pacific Arctic seawater temperatures and delayed sea ice advance in response to episodic atmospheric blocking

**DOI:** 10.1038/s41598-020-77488-y

**Published:** 2020-11-27

**Authors:** Tsubasa Kodaira, Takuji Waseda, Takehiko Nose, Jun Inoue

**Affiliations:** 1 Graduate School of Frontier Sciences, The University of Tokyo, Kashiwa, Japan; 2 Arctic Environment Research Center, National Institute of Polar Research, Tachikawa, Japan

**Keywords:** Ocean sciences, Physical oceanography, Atmospheric dynamics

## Abstract

Arctic sea ice is rapidly decreasing during the recent period of global warming. One of the significant factors of the Arctic sea ice loss is oceanic heat transport from lower latitudes. For months of sea ice formation, the variations in the sea surface temperature over the Pacific Arctic region were highly correlated with the Pacific Decadal Oscillation (PDO). However, the seasonal sea surface temperatures recorded their highest values in autumn 2018 when the PDO index was neutral. It is shown that the anomalous warm seawater was a rapid ocean response to the southerly winds associated with episodic atmospheric blocking over the Bering Sea in September 2018. This warm seawater was directly observed by the R/V Mirai Arctic Expedition in November 2018 to significantly delay the southward sea ice advance. If the atmospheric blocking forms during the PDO positive phase in the future, the annual maximum Arctic sea ice extent could be dramatically reduced.

## Introduction

Near-surface Arctic air temperatures have warmed faster than the global average by a factor of two or more in recent decades, which is a robust phenomenon known as Arctic amplification^1–3^. Global consequences of the rapid Arctic warming are anticipated because Arctic warming influences the general atmospheric and oceanic circulations and extreme weather events at mid-latitudes^2,4–7^. Despite the remarkable signal of Arctic warming, understanding of its mechanisms remains incomplete^4^. The recent rapid reduction of the Arctic sea ice extent is also concerning because of the wide-ranging impacts such as changes in the air-sea interactions^8–10^. Their adverse effects on marine mammals and other species that depend on the presence of sea ice for their survival were also reported^11,12^.

The ice-albedo feedback has been identified as the leading driver of Arctic amplification because of the significant increase in solar heating associated with the transition of albedos between the multiyear ice cover (> 0.6) and open water (∼0.07)^13–17^. Another important factor for Arctic warming is low-level clouds that cause downward longwave radiation even in winter when solar radiation is absent^18–20^. A positive feedback mechanism between the turbulent heat flux from the ocean and the downward longwave radiation from the lower troposphere was also recently reported^21^. Other factors include remote forcing mechanisms such as atmospheric heat, oceanic heat, and atmospheric moisture transports to the Arctic region from lower latitudes^22–28^. In addition to these individual factors, interactions between multiple processes are particularly important for understanding the highly nonlinear Arctic climate system^29^.

Arctic sea ice extent is one of the most important factors for the Arctic climate, and it is strongly influenced by the Arctic Ocean as well as the atmosphere. The Arctic Ocean is similar to the Mediterranean Sea because exchanges of water with adjacent oceans are crucially important^30^. With respect to seawater transport and related heat inflow to the Arctic Ocean, water volumes of 9 Sv with 28–48 TW of heat content from the Atlantic Ocean, and 1 Sv with 12 TW from the Pacific Ocean were reported^26,31,32^. Although the advective heat flux from the Pacific Ocean to the Arctic Ocean is smaller compared to the heat flux from the Atlantic Ocean, it is influential on the Arctic sea ice extent because the Pacific waters enter at depths closer to the surface than the Atlantic waters^33^.

The Pacific water enters the Arctic Ocean through the shallow and narrow Bering Strait, flows across the shallow Chukchi Sea, and intrudes to the subsurface layer over the deep western Arctic basin. Significant warming of the subsurface Pacific water layer over the western Arctic Ocean in the past three decades was recently reported^34^. Unlike the Arctic basin where the warm seawater remains subsurface, the poleward oceanic heat flux has a clearer impact on the sea ice extent in the Arctic shallow marginal seas because the heat directly acts on sea ice. The impact of the seawater temperature anomalies on the sea ice cover in early winter (November–December) was previously reported for the Barents and Bering seas^27,28^. It is also possible that the Pacific water that flows into the Beaufort Sea triggers a catastrophic sea ice reduction in the western Arctic basin through a positive feedback mechanism that significantly enhances the wind-driven sea ice export^22^.

This study focuses on the Pacific water inflow to the Arctic Ocean in relation to the Arctic sea ice advance. The area of interest is the Chukchi Sea where significant seawater warming trends, reductions of the seasonal sea ice cover, and high primary production including massive phytoplankton blooms under the sea ice were previously reported^15,35–42^. The sea ice covered period over the Chukchi Sea was recently found to be highly correlated with the oceanic heat inflow from the Pacific Ocean through the Bering Strait^43,44^. The heat transport through the Bering Strait is highly variable and complex as it depends on both the volume transport and the Pacific water temperature^45^. The volume transport depends on local winds in the Bering Strait and pressure head difference between the Arctic and the Pacific Ocean. A recent study found that the pressure difference is largely driven from the Arctic side, specifically by the zonal winds in the East Siberian Sea^46^. Serreze et al.^45^ conducted case studies for selected months and demonstrated that each factor above contributes to the Bering Strait heat inflow.

The present study uses various types of information to comprehensively evaluate the interannual variation of the Pacific Arctic Ocean for months of sea ice formation. The possible factors and consequences associated with the highest sea surface temperature (SST) in autumn 2018 are next investigated in detail. Satellite observations of SST made by the Advanced Microwave Scanning Radiometer for Earth Observing System (AMSR-E) and the Advanced Microwave Scanning Radiometer 2 (AMSR2) since 2002 (hereafter abbreviated as AMSR) were used to analyze the interannual variation of the Pacific Arctic SST. Atmospheric reanalysis data from ERA5^47^ since 1979 was then used to investigate causal factors of positive SST anomalies in autumn 2018. Direct observations near the marginal ice zone (MIZ) in the Chukchi Sea during the R/V Mirai Arctic Expedition were then combined with the other types of information to show that the warm seawater delayed the sea ice advance.

## Results

### Episodic atmospheric blocking high system in September 2018

**Figure 1:**
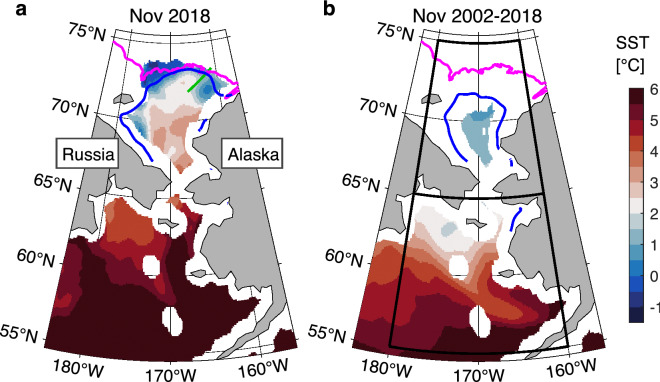
Ocean and sea ice conditions over the Pacific Arctic region in the sea ice freezing season during 2002–2018. (**a**) Monthly mean sea surface temperature (color) and 15% sea ice concentration (blue line) in November 2018. The green line is the marginal ice zone (MIZ) transect observation line. The 100 m water depth is shown by magenta line. (**b**) Same as (**a**) but for the monthly climatology of November 2002–2018. The northern and southern areas enclosed by black lines in (**b**) are used for the spatial average over the Chukchi and Bering seas, respectively. The maps are created by the MATLAB_R2020a with the mapping package M_MAP v1.4 h using the m_coast function (http://www.eoas.ubc.ca/~rich/map.html).

**Figure 2:**
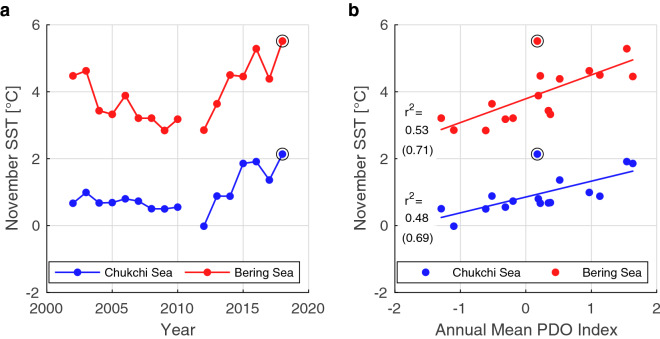
Interannual variation of November SST in the Chukchi and Bering seas and their relationships with the PDO. (**a**) November SST in the Chukchi (blue) and Bering (red) seas from AMSR satellite measurements. The black circles show the values for 2018. The SST values were spatially averaged over the areas as defined in Fig. 1b. The data in 2011 are missing because neither AMSR-E nor AMSR2 operated from September 2011 to October 2012. (**b**) November SST values plotted with the corresponding annual mean PDO index values of each year. The solid lines show the linear regression with the *r*^2^ values. The values in parenthesis are the cases excluding 2018.

Sea ice normally freezes up in November over the Chukchi Sea, but in 2018, the seawater was anomalously warm and there was less sea ice (Fig. 1). Based on satellite measurements of SST between 2002 and 2018, the November monthly SST over the Chukchi and Bering seas were their highest recorded values in 2018. Most of seawater in the Chukchi Sea originates from the Pacific Ocean, so the significant factors associated with the interannual variations of the Pacific Ocean were investigated. The November monthly SSTs varied to some extent in phase with the Pacific Decadal Oscillation (PDO) (Fig. 2). For both the Chukchi and the Bering seas, the correlation coefficients between the November monthly SST and annual PDO index were approximately 0.7 with the p-values less than 0.05 (Fig. 2b). The PDO index represents the large-scale and long-term SST variation in the North Pacific, and its positive phase corresponds to the warming in the study area^48,49^. However, the PDO is unlikely to be the cause of the exceptionally high SST in November 2018, because the PDO index was close to zero in 2018. The correlation coefficient between the PDO and SST becomes even higher if 2018 is removed (Fig. 2b), which indicates the high monthly SST in November 2018 was likely caused by other factors.

**Figure 3:**
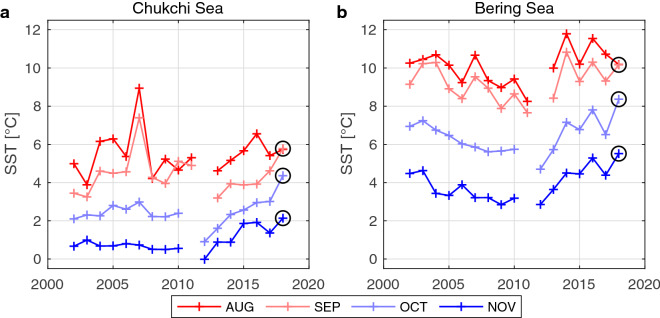
Interannual variation of the monthly sea surface temperature over the Pacific Arctic region for August to November. Panel (**a**) is for the Chukchi Sea, and (**b**) is for the Bering Sea. The black circles indicate the 2018 values. The areas for the spatial average of the Chukchi and Bering seas are shown in Fig. 1b.

AMSR satellite measurements captured rare monthly SST conditions in the Chukchi and Bering seas in 2018 (Fig. 3). The monthly SST values were almost unchanged from August to September in 2018, although the historical SST values usually decrease by several degrees during that time of the year (Fig. 3). The stable temperature was also recorded by an Argo float that was operating in the central Chukchi Sea at that time in 2018 (Supplementary Fig. 1). Previous studies indicated that the Pacific water takes at least three months to travel from the Bering Strait to the mouth of the Barrow canyon.^50,51^ This indicates that the warm Chukchi Sea observed in November 2018 can be linked to the high seawater temperatures that were present in September 2018.

**Figure 4:**
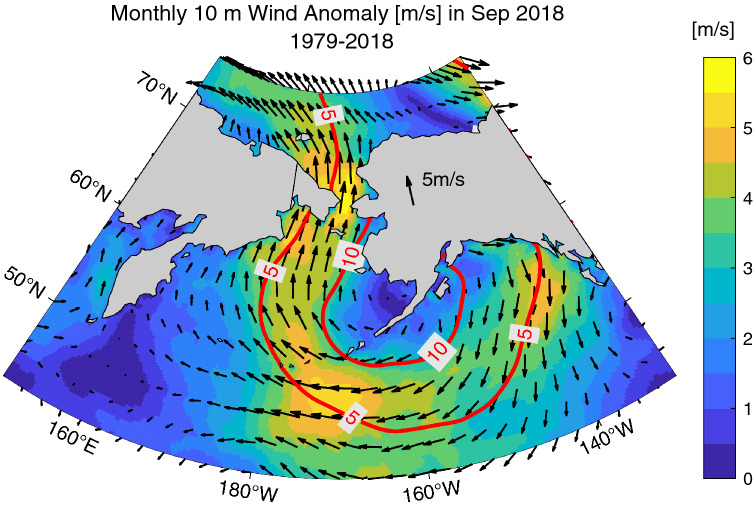
Near-surface atmospheric condition over the Pacific Arctic region in September 2018 resolved in the atmospheric reanalysis ERA5. Monthly wind anomaly (color) and monthly sea level pressure anomaly [hPa] (red lines) are shown. The anomalies were calculated with respect to the mean values during 1979–2018. The map is created by the MATLAB_R2020a with the mapping package M_MAP v1.4 h using the m_coast function (http://www.eoas.ubc.ca/~rich/map.html).

In September 2018, a distinct southerly wind anomaly occurred over the Bering Strait as a result of an unusual atmospheric condition near Alaska (Fig. 4). Based on the atmospheric reanalysis of ERA5 data, the southerly wind anomaly was approximately 5 m/s over the Bering Strait. The 5 m/s southerly wind speed anomaly corresponds to 0.70 Sv increase of the transport through the Bering Strait based on the balance between the wind stress and the sea bottom friction^26^. This additional wind-driven transport is considerable compared to the annual (September) mean transport of 1.03 Sv (0.99 Sv) calculated for 2003–2015^26^. In addition, the Bering Sea SST has increased as a result of wind-driven warm water advection. Therefore, if examined with the simple one-dimensional advection equation of seawater temperature ($\partial T/\partial t+\partial \left(VT\right)/\partial y=0$), the unusual wind pattern likely enhanced both $\Delta V\times {T}_{b}$ and ${V}_{b}\times \Delta T$ to increase Bering Strait heat inflow, where $V$ is Bering Strait transport, $T$ is Bering Sea SST, $\Delta$ is the change from the reference level, and the subscript *b* is the reference value such as climatology.

**Figure 5:**
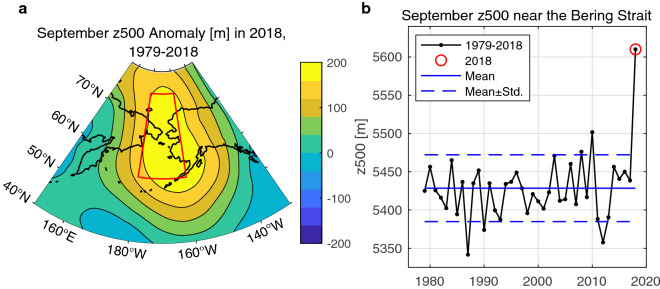
(**a**) The atmospheric blocking high system that formed over the Bering Sea in September 2018. The anomaly of the geopotential height at 500 hPa pressure level (z500) was calculated from the ERA5 dataset for September of 1979–2018. (**b**) The time series of September monthly z500 averaged over the study area enclosed by the red lines in (**a**) for the period of 1979–2018. The red circle indicates the year 2018. The blue solid line indicates the climatological mean from 1979–2018. The blue dashed line indicates one standard deviation above or below the long-term mean. The map is created by the MATLAB_R2020a with the mapping package M_MAP v1.4 h using the m_coast function (http://www.eoas.ubc.ca/~rich/map.html).

Analysis of ERA5 data showed that the unusual wind pattern in September 2018 was caused by a persistent atmospheric blocking high system in the upper troposphere over the Bering Sea (Fig. 5a). At the 500 hPa pressure level, there was positive geopotential height anomaly in the central part of the blocking high (Fig. 5a). The area-averaged geopotential height near the Bering Strait in September 2018 was approximately 180 m above the September climatology calculated as the mean from 1979–2018 (Fig. 5b). The anomaly was larger than the four standard deviations above the long-term mean. The positive pressure anomaly extended to the surface and corresponded to the near-surface southerly wind pattern (Fig. 4). This region along western Alaska can be categorized as the typical place for Pacific blocking^52,53^. The frequency of Pacific blocking is, however, significantly lower in the summer months compared to the winter months^53,54^. The atmospheric blocking in September 2018 over the Bering Sea can therefore be regarded as an episodic event.

A possible relation between the atmospheric blocking high system over the Bering Sea and the PDO was also investigated. We calculated the point correlations between the monthly z500 and PDO and found the maximum value was at most 0.42 (Supplementary Fig. 2). In addition, the spatial distribution would not explain the southerly wind pattern near the Bering Strait in September 2018. These results are consistent with the previous study that reported the Alaskan blocking is not in phase with PDO^52^. It is reasonable to think that the atmospheric blocking high system formed independently from the PDO.

**Figure 6:**
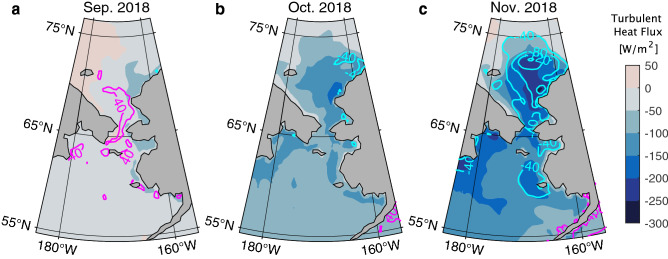
Turbulent ocean surface heat flux over the Chukchi and Bering seas based on ERA5. (**a–c**) The monthly mean heat flux [W/m^2^] (color) in September, October, and November 2018. The magenta (cyan) lines indicate the positive (negative) monthly anomaly from the mean from 1979–2018. The surface heat flux is defined as positive downwards. The turbulent heat flux is calculated as the summation of sensible heat flux and latent heat flux. The maps are created by the MATLAB_R2020a with the mapping package M_MAP v1.4 h using the m_coast function (http://www.eoas.ubc.ca/~rich/map.html).

With respect to the surface heat flux from the atmosphere to the ocean, the blocking high system in September 2018 caused positive (downwards) anomalies of sensible and latent heat fluxes over the Chukchi Sea (Fig. 6a). The positive anomalies were, however, mostly cancelled by the negative anomaly of latent heat flux in October (Fig. 6b). The variations in net short-wave and long-wave radiation were less significant (Supplementary Fig. 3). The resulting net heat flux anomaly over the Chukchi Sea from August to October 2018 was on average 2.2 W/m^2^. This corresponds to a seawater temperature increase by only 0.1 ℃ if a 40 m thick surface mixed layer was present. The minor contribution of the air-sea heat flux in 2018 indicates that the wind stress is likely the primary cause of the observed anomalous warm Chukchi Sea and the delayed sea ice freeze up in 2018. In contrast, the warmest September SST on record rapidly cooled down in October 2007 (Fig. 3). In October 2007, the relatively strong sustained easterly off-ice wind was present in the northern part of the Chukchi Sea, but such an atmospheric condition was not present in 2018.

### Anomalously warm Chukchi Sea that delayed sea ice advance in November 2018

The effects of the anomalous warm seawater in the Chukchi Sea on sea ice formation were examined based on the results of R/V Mirai Arctic Expedition in November 2018. The intensive hydrographic observational results near the MIZ during 5–9 November 2018 indicate the warm Pacific water formed an approximately 40 m thick surface mixed layer over the shallow Chukchi Sea (Supplementary Fig. 4). The heat content stored in the mixed layer was sufficiently large to delay the sea ice advance for 74 days if the surface heat flux remained constant at − 94 W/m^2^, which was the November climatology based on the ERA5 reanalysis. A similar warm surface layer was also present across wider areas of the Chukchi Sea^55^.

**Figure 7:**
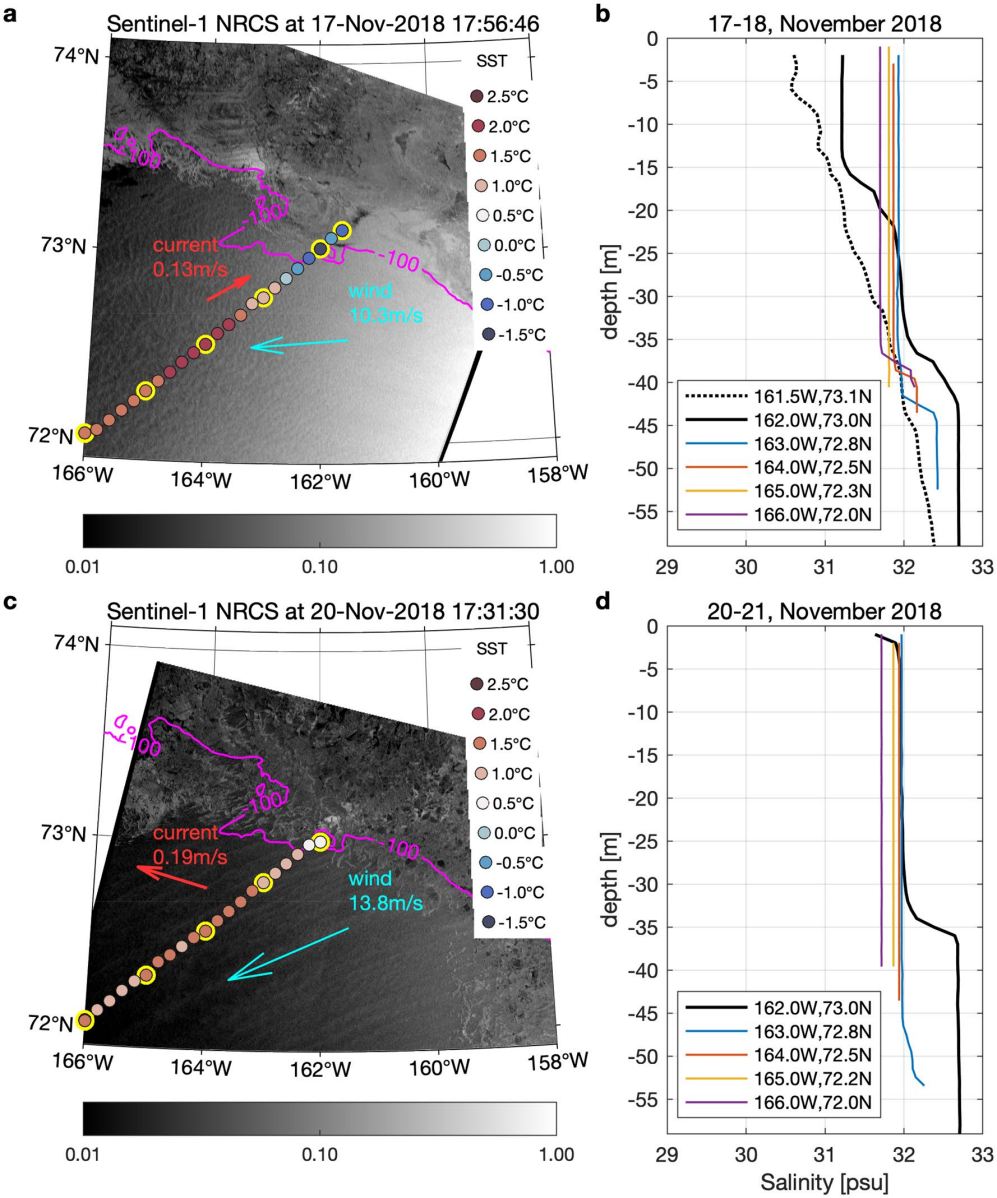
Sea ice, ocean, and atmosphere variations over the northeastern Chukchi Sea during the R/V Mirai cruise in November 2018. (**a**) Normalized Radar Cross Section (NRCS) from Synthetic Aperture Radar (SAR) on Sentinel-1 on 17 November. The relatively large NRCS area near and north of the 100 m isobath (magenta line) indicates increased radar backscattering by the presence of the sea ice. The magenta line shows the 100 m isobath to indicate the shelf edge. The colored dots show seawater temperature at 5 m. The red and blue arrows show in-situ currents averaged over 8–32 m below the sea surface and wind vectors at 25 m above sea level, respectively. The seawater temperature and wind data are the spatial averages of the results during the cruise transect (Supplementary Figs. 5b and 6a). The data from the 24 h with the SAR measurement occurring at the central time are used. (**b**) Salinity profiles were obtained at the yellow outlined points shown in (**a**) on 17–18 November. (**c**, **d**) are the same as (**a**, **b**), respectively, but for 20–21 November. The maps are created by the MATLAB_R2020a with the mapping package M_MAP v1.4 h (http://www.eoas.ubc.ca/~rich/map.html).

R/V Mirai conducted unique daily hydrographic and atmospheric measurements along a fixed transect toward the sea ice edge during 9–21 November 2018 to observe variations of air-wave-ice-ocean conditions in the MIZ (see, the green line in Fig. 1 and Supplementary Fig. 4). The continuous measurements included near-surface air temperature and wind, sea surface temperature and salinity, and horizontal current (Supplementary Figs. 5–7). The most notable event was that there were sustained below-freezing, off-ice northeasterly wind conditions during 17–21 November (Supplementary Fig. 5). The wind speed of the cold-air outbreak was over 10 m/s, and the air temperature was often below − 10 °C near the sea ice. Despite the ideal freezing conditions, the unusually warm water prevented sea ice advance. The freezing wind generally decreased the seawater temperature, but the temperature was still much higher than the freezing point of − 1.8 °C. As a result, the sea ice advance was minimal during this off-ice wind condition period. The slowing of sea ice advance during 17–21 November (Fig. 7a,c) is obvious if compared with the sea ice advance of 30–50 km during 9–12 November under the moderate northerly winds (Supplementary Figs. 5b and 8).

**Figure 8:**
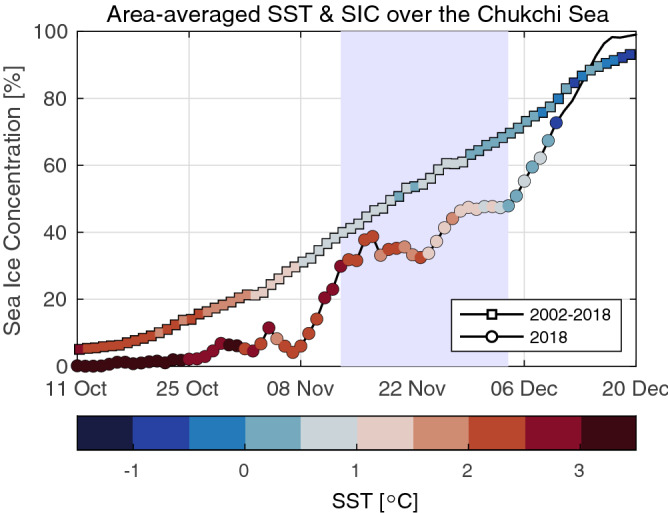
Time series of sea ice concentration and sea surface temperature (colored dots) from the AMSR satellite measurements, averaged over the Chukchi Sea (see, Fig. 1b). The black line with squares is for the mean during 2002–2018 and the black line with circles is for 2018.The blue shaded area indicates the period, 13 November–4 December, when the sea ice advance was remarkably delayed.

Interestingly, the SST near the MIZ on 20 November was even slightly warmer compared to 17 November (Fig. 7a,c), despite the large heat release expected during the intense freezing off-ice wind conditions. A possible surface warming mechanism could be upward heat transfer by oceanic vertical mixing caused by strong winds and waves^56^. An alternative mechanism is the horizontal advection of warm water. The ocean currents measured by the shipborne Acoustic Doppler Current Profiler (ADCP) support the possibility of horizontal warm water advection. During the MIZ transect observation period of 9–21 November 2018, the eastward currents near the MIZ gradually veered northeastward until the current eventually turned westward on 20 November (Supplementary Fig. 7). The sustained ~ 0.12 m/s eastward currents over the 10 days indicate that the integrated transport distance of the warm water corresponds to 100 km. The observed warm seawater near the MIZ was likely advected from the central Chukchi Sea where relatively high SST was detected (Fig. 1). Another support for this is that the near-surface profiles of salinity at 162°W matched with those along 163°W on 21 November, with a difference of less than 0.1 psu (Fig. 7b,d). Assuming salinity as a tracer, the agreement indicates there was northeastward horizontal advection of warm Pacific water along the MIZ transect. These facts indicate that the warm water advection likely increased the SST near the MIZ.

Satellite measurements of the sea ice concentration (SIC) provided additional information about the delay of the sea ice advance around the broader area of the Chukchi Sea. Compared to the mean during 2002–2018, SIC was lower from October 2018 and the SST was higher (Fig. 8). In 2018, SIC started increasing rapidly after 8 November, which indicates the southward sea ice advance from the central part of the Arctic to the Chukchi Sea. The advance of the sea ice, however, was significantly slowed from 13 November to 4 December 2018 (Fig. 8). SIC changed very slowly during that period, while the seawater temperature decreased significantly by approximately 3 °C by the anomalous turbulent surface heat flux (Supplementary Fig. 3a,b). These changes indicate that the sea ice advance was prevented by the warm water over the shelf until the oceanic heat was fully released into the atmosphere.

In November 2018, the heat release from the Chukchi Sea to the atmosphere was significantly higher than the monthly mean from 1979–2018 (Fig. 6c and Supplementary Fig. 3). This larger air-sea heat flux was caused by the unusually warm and open Chukchi Sea because the upward turbulent fluxes can be significantly increased. Since the atmospheric condition in November 2018 was more favorable for sea ice formation than the previous years, the slowing of the sea ice advance in November 2018 appears to have been primarily caused by the anomalously warm Chukchi Sea.

## Discussion

The anomalous warm and open Chukchi Sea in late autumn 2018 was studied in the context of interannual variation using a variety of data. We found that seawater temperature in the Chukchi Sea in the sea ice freezing season was highly correlated with the PDO index in the last two decades. However, the highest autumn seasonal values of SST in the Chukchi Sea were recorded in 2018 when the PDO index was close to zero. The primary factor causing the anomalous warm conditions in the Chukchi Sea was found to be the episodic blocking high pressure system over the Bering Sea in September 2018. The blocking high caused an unusual near-surface southerly wind pattern near the Bering Strait. As a result, warm Pacific seawater intruded into the Chukchi Sea in September 2018 and subsequently delayed sea ice advance in November 2018.

Atmospheric blocking over the northern North Atlantic was previously reported to influence the Atlantic multidecadal ocean variability^57^. More recently, the North Atlantic atmospheric blocking was found to have an additional role in modulating sea ice export through the Fram Strait and eventually impacts the Atlantic Meridional Overturning Circulation^58^. However, an impact of the atmospheric blocking on the autumn sea ice growth over the Pacific Arctic region has never been reported. This study presented the first evidence that the atmospheric blocking over the Pacific Arctic region plays a significant role in the modulation of the Pacific Ocean inflow to the Arctic Ocean and its impact on the sea ice advance.

We further examined whether PDO has a strong effect on the Chukchi Sea SST for months other than November. The correlation coefficient between the monthly averaged Bering Sea SST and the annual PDO index was larger than 0.7 for most of the months (Supplementary Fig. 9). For the Chukchi Sea, however, the correlation was spotty as it was previously reported with the sustained in-situ measurement results at the Bering Strait^45^. The low correlation in the summer months implies the other factors are influential such as the Bering Strait volume transport and the ocean surface heat flux over the Chukchi Sea. These two factors are strongly influenced by the atmospheric condition over the Pacific Arctic region^45^. The condition can be in part described by climate indices such as the Arctic Oscillation index. The potential factors could be however more diverse as this study showed that the single atmospheric blocking event had a significant influence on the Chukchi Sea SST condition from September to November.

The elevated SST and delayed sea ice formation reported in this study have widespread impacts over the Pacific Arctic region and beyond. For example, a recent study showed that unusual mortality of tufted puffins occurred in the eastern Bering Sea in October 2016 through January 2017^59^. Since the shifts in the zooplankton community and forage fish distribution followed a period of elevated sea surface temperature^59^, a bottom-up shift in seabird prey availability is possible. Decreasing SIC also affects species like polar bears and seals that depend on the ice being present each year^11,12^. The longer ice-free periods also permit Pacific water to transport heat to the central part of the Arctic basin^60,61^. An accurate assessment of the transports and their consequences on the entire western Arctic requires a better understanding of the turbulent flux by oceanic eddies in addition to the advective flux. The oceanic eddies are often observed near the shelf break, and also near the MIZ, as observed by the Sentinel-1A/B during the MIZ transect observations of the R/V Mirai in October 2018^42,62^ (see Supplementary Fig. 8). These eddies create an important pathway of water exchange between the Chukchi Sea and the Arctic basin, which requires further study.

A better representation of the oceanic heat transport from the Pacific to the Arctic is also important for safe ship navigation over the sea ice covered Arctic Ocean. The ships in the Arctic can encounter hazards such as collision with perennial sea ice, sea-spray icing, and high winds and waves during cyclones^63^. A recent study also showed correlations between wind speeds and increases of wave heights in the Arctic Ocean from 1979 to 2016^64^. Accurate representations of the sea ice distribution were recently found to be an important factor for making daily wave forecasts^65^, which are of interest to the Arctic shipping community. These studies indicate that the findings of this study about the factors and consequences of the Pacific heat inflow may be able to facilitate better prediction of the sea states and safe ship navigation in the Arctic.

The delayed sea ice advance over the Pacific Arctic region can also be influential even on mid-latitude atmospheric conditions. The linkage between the anomalous Arctic warming near Alaska and the cold winters in eastern North America during 2013/2014 and 2014/2015 was previously discussed^66^. More recently, it was shown that jet stream pathways can be changed by the large upward heat flux from the open Chukchi Sea in the early winter^67^. Consequently, the atmospheric thermal front moves southward and causes cold winters in Asia and North America. The small sample of years with a large sea ice loss, however, makes it difficult to clarify the causal relation between the sea ice decline and the changes in mid-latitude weather systems^68^.

This study showed that the evolution of the atmospheric blocking is independent of PDO, but the blocking high system can form during the PDO positive phase in the future. The emergence of the blocking high system in summer seasons over the Bering Sea is considered to be rare event even during the rest of the twenty-first century^54^. However, from a statistical point of view, atmospheric blocking will eventually form during the PDO positive phase in the future. The regression analysis indicates the Chukchi Sea SST increased by approximately 1 °C in 2015 when the PDO index was at a maximum (Fig. 2). The results also indicate that the Chukchi Sea SST response in November to the atmospheric blocking in September 2018 was slightly higher than 1 °C. If the atmospheric blocking forms during the PDO positive phase in the future, the SST could increase more than 2 °C and dramatically reduce the annual maximum Arctic sea ice extent. Careful future monitoring of the atmospheric conditions in late summer will be important for seasonal prediction of the sea ice extent in winter, which is concerning at both the local and global scales during this period of global warming.

## Methods

### SST and SIC over the Chukchi and Bering seas

Sea surface temperature (SST) distribution over the Chukchi and Bering seas was mapped using a daily 1/10° resolution SST dataset that was created from measurements of the Advanced Microwave Scanning Radiometer for Earth Observing System (AMSR-E) and the Advanced Microwave Scanning Radiometer 2 (AMSR2). Daily sea ice concentration (SIC) distribution based on AMSR-E and AMSR2 measurements was also used with 1/10° resolution. The analysis period was from 2002 to 2018, but the observations in part of 2011 and 2012 are missing because the AMSR-E data are available from 2002 to September 2011, and the AMSR2 replaced the AMSR-E for the observations since October 2012. Daily SST and SIC data were downloaded from the Arctic Data archive System (ADS) at http://ads.nipr.ac.jp/.

The monthly averaged spatial distributions of SST and SIC were first created by taking the temporal mean without including missing data. The spatially averaged SST and SIC for the Chukchi Sea and the northern Bering Sea were further calculated as the mean over the areas 65–75˚N, 160–180˚W and 55–65˚N, 160–180˚W, respectively (see, Fig. 1b). The SST values near the sea ice covered areas and coastal areas are missing and not included when the spatial average is calculated.

### Decadal variation of North Pacific SST

Decadal variation of North Pacific SST is represented by the Pacific Decadal Oscillation (PDO) Index. PDO index is statistically defined as the leading principal component of North Pacific monthly sea surface temperature variability (poleward of 20°N). The PDO index values data were downloaded from the website of Dr. Mantua at http://research.jisao.washington.edu/pdo/. The annual mean values were calculated using the mean monthly values. Since the index was available up to September 2018 on the website, the annual mean value for 2018 was calculated as the mean from January to September.

### Atmospheric conditions over the Pacific Arctic and North Pacific

Estimates of winds and air-sea heat flux over the study area were made for 1979–2018 with ERA5 reanalysis data produced by the European Centre for Medium-Range Weather Forecasts (ECMWF). The total air-sea heat flux was calculated as the sum of surface latent heat flux, surface sensible heat flux, surface net solar radiation, and surface net thermal radiation. Geopotential height anomalies at 500 hPa levels were used to identify the atmospheric blocking following the study of Dole and Gordon^69^. Mean sea level pressure anomalies were also used to discuss the atmospheric blocking over the study area. The monthly mean data were obtained from the ECMWF data server (http://apps.ecmwf.int/datasets/), with a spatial resolution of 0.25°.

### Argo float in the central Chukchi Sea in 2018

The Argo data were collected and made freely available by the International Argo Program and the national programs that contribute to it. (http://www.argo.ucsd.edu, http://argo.jcommops.org). The Argo Program is part of the Global Ocean Observing System. The vertical profiles of seawater temperature and salinity were calculated from Argo float #4902926 that drifted within the central Chukchi Sea in 2018 at water depths of 40–60 m to study the seasonal evolution of the mixed layer depth and upper ocean heat conditions.

### R/V Mirai Arctic expedition in 2018

R/V Mirai entered the Chukchi Sea from the Bering Strait on 4 November 2018 to conduct observations of atmosphere, ocean, waves, and sea ice during the R/V Mirai cruise MR18-05C. This Arctic expedition was approximately three weeks and finished on 24 November 2018 by passing out of the Bering Strait. During the expedition, the near-surface atmospheric and oceanographic variables were continuously measured by the Shipboard Oceanographic and Atmospheric Radiation (SOAR) measurement system^70^. The air temperature and wind were measured at 25 m using the anemometer (Model 05106, R.M. Young), while the seawater temperature and salinity were measured at 5 m below the sea surface using an SBE-45, (Sea-Bird Electronics, Inc.). The 5-min moving averages were applied for the visualizations. The shipboard ADCP (75 kHz, RDI) was used to measure the ocean currents. The 30-min averages were applied for the visualizations. These data can be obtained from the Data and Sample Research System for Whole Cruise Information in JAMSTEC (http://www.godac.jamstec.go.jp/darwin/cruise/mirai/mr18-05c/e). Temperature, conductivity, depth (CTD) measurements were conducted with an SBE9plus (S/N 09P54451-1027, Sea-Bird Electronics, Inc.). Following previous studies^56,71^, the oceanic mixed layer depth was calculated as the shallowest depth at which potential density is more than 0.1 kg/m^3^ higher than the near-surface mean potential density, and the near-surface mean potential density was calculated as the average from 5 to 10 m below the sea surface.

### The sea ice extent from SAR images

The Level-2 SAR data of the normalized radar cross-section from Sentinel-1A/B were used to estimate the sea ice extent near the study area. The data were linearly interpolated to the regular longitude and latitude grids with 1/500° resolution for the visualizations. The data were created by NOAA and were obtained from NOAA CoastWatch (http://coastwatch.noaa.gov). The original data of the Sentinel-1A/B are provided to NOAA by the Copernicus Program.

## Supplementary information

Supplementary Information 1.

## Data Availability

All new data that support the findings of this study can be provided upon a request by the time of possible publication. At the time of publication, the data can be fully accessed via the Arctic Data archive System, managed by the Research Organization of Information and Systems, Polar Environment Data Science Center, National Institute of Polar Research, Japan.

## Code availability

All the procedures were completed using custom MATLAB scripts, which are available upon request.
