# Supplementary material for: Record high Pacific Arctic seawater temperatures and delayed sea ice advance in response to episodic atmospheric blocking
Source: Sci Rep. 2020 Nov 27;10:20830. doi: 10.1038/s41598-020-77488-y (PMC7695746; doi:10.1038/s41598-020-77488-y)
Supplement: Supplementary file 1 — Supplementary Information 1. [file 41598_2020_77488_MOESM1_ESM.docx]

**Supplementary Material**

*Scientific Reports*

Record high Pacific Arctic seawater temperatures and delayed sea ice advance in response to episodic atmospheric blocking

Tsubasa Kodaira*^1^, Takuji Waseda^1^, Takehiko Nose^1^ and Jun Inoue^2^

^1^ Graduate School of Frontier Sciences, the University of Tokyo, Japan.

^2^ Arctic Environment Research Center, National Institute of Polar Research, Japan.

Address for correspondence: [kodaira@edu.k.u-tokyo.ac.jp](mailto:kodaira@edu.k.u-tokyo.ac.jp))

**List of contents**

1. **Supplementary Figures**.

**Supplementary Fig. 1**: Temporal variation of the seawater temperature over the central Chukchi Sea based on Argo float #4902926.

**Supplementary Fig. 2**: The spatial distribution of the correlation coefficient between the PDO index and z500 September monthly anomaly.

**Supplementary Fig. 3**: Area-averaged sensible heat flux, latent heat flux, net short-wave radiation flux, and net long-wave radiation flux over the Chukchi Sea.

**Supplementary Fig. 4**: Sea surface temperature (SST), Mixed layer depth (MLD), and sea-ice concentration (SIC) over the northeastern Chukchi Sea, 5-9 November 2018.

**Supplementary Fig. 5:** Near surface atmospheric conditions from R/V Mirai along the ship track during the MIZ transect observations.

**Supplementary Fig. 6**: Near surface ocean conditions from R/V Mirai along the ship track during the MIZ transect observations.

**Supplementary Fig. 7:** Near surface ocean currents from R/V Mirai during the MIZ transect observation.

**Supplementary Fig. 8:** Sea ice, ocean, and atmosphere variations over the northeastern Chukchi Sea during the R/V Mirai cruise in November 2018.

**Supplementary Fig. 9**: Correlation coefficient between the monthly SST over the Chukchi and Bering seas and annual PDO index for the period of 2002-2018.

Supplementary Fig. 1: Temporal variation of the seawater temperature over the central Chukchi Sea based on Argo float #4902926. a The Argo float trajectory in Aug-Nov 2018. The black lines show bathymetry [m]. b Temperature profiles obtained by the Argo float. The black line shows the mixed layer depth. The map is created by the MATLAB_R2020a with the mapping package M_MAP v1.4 h using the m_coast function (https://www.eoas.ubc.ca/~rich/map.html).

Supplementary Fig. 2: The spatial distribution of the correlation coefficient between the PDO index and z500 September monthly anomaly. The map is created by the MATLAB_R2020a with the mapping package M_MAP v1.4 h using the m_coast function (https://www.eoas.ubc.ca/~rich/map.html).

Supplementary Fig. 3: Area-averaged sensible heat flux, latent heat flux, net short-wave radiation flux, and net long-wave radiation flux over the Chukchi Sea. The square marked with the error bars indicate the mean and standard deviation from 1979-2018. The black circles are for 2018. The area for the average is 65-75˚N, 160-180˚W (see, Fig.1b).

Supplementary Fig. 4: Sea surface temperature (SST), mixed layer depth (MLD), and sea-ice concentration (SIC) over the northeastern Chukchi Sea, 5-9 November 2018. SST data in color are from the AMSR2 and the R/V Mirai measured at -5 m along the track (black line). The color of large circles shows the temperature from CTD casts, on average 1.8 m below the sea surface. The text inside the large circles shows MLD. The direction of R/V Mirai can be found by the dates in the cyan box. The ice-covered area (SIC>15%) is the white area in the upper right enclosed by the blue line. The green line indicates the MIZ transect. The map is created by the MATLAB_R2020a with the mapping package M_MAP v1.4 h using the m_coast function (https://www.eoas.ubc.ca/~rich/map.html).

Supplementary Fig. 5: Near-surface atmospheric conditions from R/V Mirai along the ship track (green line in Supplementary Fig. 4) during the MIZ transect observations. a Air temperature and b wind speed. The black arrows in b indicate the daily mean wind vector.

Supplementary Fig. 6: Near-surface ocean conditions from R/V Mirai along the ship track (green line in Fig.2) during the MIZ transect observations. a temperature and b salinity. The white circles indicate CTD cast locations.

Supplementary Fig. 7: Near-surface ocean currents from R/V Mirai (green line in Supplementary Fig. 4) during the MIZ transect observations. a Current speed and b current direction averaged over 162°W-163°W. The currents are averaged over the depth averaged over 8-32 m. The current direction of 0° is northward and changes clockwise as 90°is eastward. The data within the shaded area in a were used to create b. The red line indicates direction of the MIZ transect.

Supplementary Fig. 8: Sea ice, ocean, and atmosphere variations over the northeastern Chukchi Sea during the R/V Mirai cruise in November 2018. a Normalized Radar Cross Section (NRCS) from Synthetic Aperture Radar (SAR) on Sentinel-1 on 9 November. The relatively large NRCS area near and north of the 100 m isobath (magenta line) indicates increased radar backscattering by the presence of the sea-ice. The magenta line shows the 100 m isobath to indicate the shelf edge. The colored dots show seawater temperature at -5 m. The red and blue arrows show in-situ currents averaged over 8-32 m below the sea surface and wind vectors at 25 m above sea level, respectively. The seawater temperature and wind data are the spatial average of the results during the cruise transect (Supplementary Figs. 5 and 6). The data from the 24 hours with the SAR measurement occurring at the central time are used. b is the same as a, but for 12 November. The maps are created by the MATLAB_R2020a with the mapping package M_MAP v1.4 h using the m_coast function (https://www.eoas.ubc.ca/~rich/map.html).

Supplementary Fig. 9: Correlation coefficient between the monthly area averaged SST and annual PDO index for the period of 2002-2018. The red and blue lines show the case results for the Bering Sea and Chukchi Sea, respectively. The area for the average is 65-75˚N, 160-180˚W (see, Fig.1b). The circle marks are added to the months when the p-values are less than 0.05.
